# Involvement of 5′ and 3′ UTRs in SARS-CoV-2 Virus-like Particle Genome Packaging

**DOI:** 10.3390/v18070700

**Published:** 2026-06-25

**Authors:** Zhang Zhang, Kun Yang, Fangze Shao, Wenlong Shen, Ping Li, Yue Zhang, Junjie Xu, Dejian Xie, Chudong Wang, Guoying Yu, Jun Zhang, Zhihu Zhao, Yan Zhang

**Affiliations:** 1 Laboratory of Advanced Biotechnology, Beijing Institute of Biotechnology, Beijing 100071, China; zzhnng@126.com (Z.Z.); sfz0603@foxmail.com (F.S.); shenw1988@163.com (W.S.); lipingtry@163.com (P.L.); zhangyue4381@126.com (Y.Z.); xujunjie@sina.com (J.X.); ncuskxiedejian@163.com (D.X.); bachelormocker@gmail.com (C.W.); 2 College of Life Sciences, Henan Normal University, Xinxiang 453007, China; yangkun@stu.htu.edu.cn

**Keywords:** SARS-CoV-2, RNA structure, virus genome, packaging

## Abstract

The molecular mechanisms governing the efficient packaging of the large SARS-CoV-2 RNA genome into progeny virions remain incompletely understood, with the role of untranslated regions (UTRs) being particularly enigmatic. Leveraging proximity ligation sequencing data, we identified direct, high-frequency interactions between the viral packaging signal PS9 and both the 5′ and 3′ UTRs during intracellular replication stages. Functional validation using an infectious virus-like particle (iVLP) system demonstrated that genomes incorporating SARS-CoV-2 UTRs exhibited significantly enhanced packaging efficiency, yielding an increase in both packaged RNA copies and reporter gene expression post-infection. Competitive packaging assays confirmed the UTRs confer a selective advantage during particle assembly. Mechanistically, Western blot and digital Western analysis revealed that UTR-containing iVLPs incorporated approximately 2-fold more nucleocapsid (N) proteins, suggesting enhanced N recruitment or retention. The deletion of specific core sequences within the UTRs predicted to form a base pair with PS9 abrogated this enhancement, suggesting the functional significance of the UTR-PS9 interaction interface. Collectively, these results establish that the 5′ and 3′ UTRs act synergistically through direct RNA-RNA interactions with PS9 to promote N protein recruitment and enhance packaging efficiency in a PS9-dependent iVLPs system. This UTR-PS9 regulatory axis presents a novel target for therapeutic intervention against SARS-CoV-2 and related coronaviruses.

## 1. Introduction

The efficient packaging of genomic RNA (gRNA) into progeny virions is a fundamental step in the life cycle of coronaviruses. This process ensures that the ∼30 kb viral genome, rather than abundant cellular mRNAs or subgenomic viral RNAs (sgRNAs), is efficiently incorporated into assembling particles [1]. Coronaviruses, like many other ssRNA viruses, assemble their nucleocapsid (N) protein around the genome to form a beads-on-string ribonucleoprotein (RNP) structure [2,3,4].

It is established that this selectivity critically relies on cis-acting packaging signals (PSs) embedded within the gRNA and their interactions with viral structural proteins [5,6]. Among these, the N protein plays a central and indispensable role in the packaging process. The N protein, the major RNA-binding component of the viral nucleocapsid, possesses high capacity for nonspecific RNA binding, driven by its intrinsically disordered regions [7,8]. However, efficient condensation of the gRNA into nascent virions requires additional regulatory inputs.

Although the precise location of the packaging signal within the SARS-CoV-2 genome is a subject of ongoing debate, one prominent candidate has been identified within a region designated as “T20” (nt 20,080–22,222). This T20 could be further truncate to the “PS9” region (nt 20,080–21,171); both could promote virus assembly independently [5].

Previous studies indicate that coronavirus packaging signals not only facilitate viral genome packaging but also induce phase separation driven by the nucleocapsid (N) protein. This dual functionality has prompted investigations into the molecular mechanisms governing specific interactions between packaging signals and nucleocapsid proteins [9,10]. These studies have also proposed the potential involvement of untranslated regions (UTRs) in N protein phase separation, thereby suggesting a role for UTRs in the coronavirus packaging process. Nonetheless, direct functional evidence substantiating the participation of UTRs in this process remains elusive [11,12,13].

Leveraging proximity ligation data from our simplified SPLASH assay [14] and complementary COMRADES analysis [15], we mapped the PS9-centered RNA interactome to identify genomic elements critical for SARS-CoV-2 packaging. This approach revealed direct 5′/3′ UTR-PS9 RNA interactions during the intracellular phase, with high-frequency ligation events, indicating dynamic scaffold formation in pre-assembly stages. Functional validation demonstrated that UTRs enhance packaging efficiency and boost N protein recruitment, while truncation mutagenesis identified core UTR sequences essential for both PS9 binding and packaging enhancement.

## 2. Materials and Methods

### 2.1. Cells

The Lenti-X 293T cell line was purchased from Clontech (now TAKARA Bio, San Jose, CA, USA), while the HEK-293T-ACE2 cell line was procured from Shanghai Anwei Biotechnology, Shanghai, China. Cells were cultured in Dulbecco’s modified Eagle’s medium (DMEM, Pricella, Wuhan, China) supplemented with 10% fetal bovine serum (FBS, Pricella, Wuhan, China), 100 U/mL penicillin and 100 μg/mL streptomycin (Invitrogen, Carlsbad, CA, USA) at 37 °C in an atmosphere of 5% CO_2_.

### 2.2. Plasmid Construction and Molecular Cloning

Plasmids Luc-PS9, M+E (CoV2-M-IRES-E), GFP-PS9, N (CoV2-N-R203M), and S (CoV2-Spike-D614G) were sourced from Addgene. ULP denotes the insertion of the 5′ untranslated region (UTR) of SARS-CoV-2 at the 5′ end of Luc in LP, while LPU represents the insertion of the 3′ UTR of SARS-CoV-2 at the 3′ end of PS9 in LP. ULPU signifies the insertion of both the 5′ UTR of Luc in LP into the 5′ UTR of SARS-CoV-2 and the 3′ end of PS9 into the 3′ UTR of SARS-CoV-2. Variants such as 5UTRΔ49, 5UTRΔ105, and 3′ UTRΔ84 involve targeted nucleotide deletions while preserving crucial interaction sequences.

### 2.3. PS9 Interaction Identification

This study utilized various public datasets, including the simplified SPLASH dataset established by our laboratory [14], COMRADES data [15]. The viewpoint method was utilized in accordance with previous reports [16]. Briefly, all sequencing reads were aligned to the SARS-CoV-2 reference genome (NC_045512.2). The PS9 region was isolated, and interacting fragments across the entire genome were identified. This approach aimed to elucidate the comprehensive interactions of the PS9 region with the genome, laying the groundwork for further functional investigations. Additionally, based on the interaction’s intensity, determined by the number of supporting chimeric reads, long-range interaction bases were predicted. The Mfold algorithm was employed to predict RNA interactions, pinpointing specific paired bases.

All sequencing reads were aligned to the SARS-CoV-2 reference genome (GenBank: NC_045512.2); chimeric reads were called and annotated with the hyb package [17]. Viewpoint-based analyses were performed as previously reported [14]: each chimeric read was split and mapped to two paired, non-overlapping 10 nt bins. Interaction scores were calculated as log_2_-transformed chimeric read counts, and z-scores were computed for all bin–bin interaction pairs. Interactions with z-score > 2.13 (corresponding to log_2_ chimeric reads exceeding the mean at 95% confidence) were considered enriched. Among these, enriched interactions involving the PS9 region were selected if either arm of the interaction fell within the PS9 viewpoint. To further assess statistical significance, a differential interaction analysis was performed using DESeq2 [18], comparing chimeric read counts in 10 nt × 10 nt bin pairs between ligated and control datasets. Only interactions with adjusted *p*-value < 0.05 and log_2_ fold-change > 1 were retained for downstream analysis.

### 2.4. iVLPs Production

During the production of iVLPs, in addition to the plasmid coding structure protein of SARS-CoV-2, the iVLPs genome plasmid with or without UTRs was added in equivalent molar ratios. Plasmids were co-transfected according to the manufacturer’s application note of improved lentiviral production using lipofectamine 3000 reagent (Invitrogen, USA). For 6-well culture plates, approximately 1.6 × 10^6^ cells per well were seeded in 2 mL of lentiviral packaging medium. For 10 cm dishes, 9.6 × 10^6^ cells per dish were plated in 12 mL of lentiviral packaging medium. Plasmids, lipofectamine 3000 and P3000 reagent were added according to instructions. After 48 h, cell supernatant was harvested from each well or dish, clarified by centrifugation, and filtered using a 45 µm pore size PES filter (Pall Corporation, Port Washington, NY, USA). The clarified lentiviral supernatant was aliquoted into cryovials and stored at −80 °C.

### 2.5. Luciferase Assay

In each well of a transparent 96-well plate, 50 µL of supernatant containing iVLPs was dispensed, followed by the addition of 100 µL of antibody-free 293T cell-specific medium containing 35,000 recipient cells (HEK-293T-ACE2). The plate was then incubated at 37 °C with 5% CO_2_ for 24 h to facilitate viral entry and infection. Following the incubation period, all the culture medium was carefully aspirated from each well of the transparent 96-well plate. Subsequently, 100 µL of Bio-Lite detection reagent (Vazyme, Nanjing, China), pre-equilibrated to room temperature, was added to each well. The plate was left at room temperature for a minimum of 3 min to allow for complete cell lysis. Thereafter, 80 µL of cell lysate from each well was transferred to an opaque white 96-well plate, and luciferase luminescence was quantified using a microplate reader Infinite M200PRO (Tecan, Zurich, Switzerland) with the auto-attenuation function set and an integration time of 1000 ms.

### 2.6. Droplet Digital PCR and Competitive Packaging Experiments

Supernatants were then collected for RNA extraction from the packaged virus-like particles, and the differential transcript content was assessed using droplet digital PCR. RNA extraction from virus-like particles or cells was executed utilizing Qiagen’s RNeasy Plus Kit. Quantification of specific gene transcripts was achieved through droplet digital PCR (ddPCR) using the Digital LightCycler 5× RNA Master Kit (Roche, Basel, Switzerland) with specific primers and probes. The amplification efficiency and specificity of primer sets were determined by qPCR of a serial diluted template. In competitive packaging experiments, each iVLPs’ genome plasmid was transfected into Lenti-X 293 T cells with equivalent molar ratios and their cell lysate was collected with the iVLPs’ yield. These samples were controls for normalizing the efficiency of different plasmids’ transfection and PCR amplification. After normalization, the relative advantage of the iVLPs genome containing UTRs was calculated with the following formula: U/(L + U) × 100%. U represents the iVLPs genome containing UTRs; L represents the genome without UTRs.

### 2.7. Dual Luciferase Competition Experiment

During the packaging of iVLPs, in addition to the plasmids’ coding structure protein of SARS-CoV-2, the genome plasmid containing wildtype or truncated UTRs was added at equivalent molar ratios. Firefly luciferase was used as a reporter of the genome with wildtype UTRs, while renilla luciferase was used for truncated UTRs. Subsequent to infection experiments with the packaged iVLPs, luminescence readings were acquired using the Duo-Lite Luciferase Assay System (Vazyme, Nanjing, China).

### 2.8. Western Blotting

An appropriate volume of supernatant containing iVLPs was combined with a 1/4 volume of protein loading buffer and heated in a boiling water bath for 5 min. Post-cooling, the mixture was briefly centrifuged, and the resulting pellet was loaded onto a suitable tube. Electrophoresis was conducted on a gradient gel, followed by gel transfer to a PVDF membrane. The membrane was then blocked with a quick blocking solution and subsequently incubated with primary antibodies overnight at 4 °C. After washing with TBST buffer, the membrane was incubated with secondary antibodies for 1 h at room temperature. Chemiluminescence imaging was performed using iBright1500 (Invitrogen, Carlsbad, CA, USA).

### 2.9. Digital Western Blotting

Capillary-based Western blot (Wes) analysis was performed according to the manufacturer’s instructions for the 12–230 kDa protein separation module (#SM-W004; ProteinSimple, San Jose, CA, USA). The supernatant containing iVLPs was centrifuged at 12,000× *g* for 3 min at 4 °C, and the supernatant was collected for use. The sample was diluted 1:4 with 10× sample buffer and PBS, mixed thoroughly, heated to 95 °C for 5 min, and cooled on ice for 5 min to facilitate viral lysis. Sample preparation was carried out according to the manufacturer’s protocol, using a 5× mastermix without DTT, heated to 95 °C for 5 min, and cooled on ice for 5 min. The prepared samples were loaded onto Simple Western plates. The primary antibody to SARS Spike Protein (1:1500, NB100-56578, Novus Biologicals, Littleton, CO, USA) and SARS-CoV-2 Nucleocapsid (1:80, 40588-T62, Sino Biological, Inc., Beijing, China) was diluted in ProteinSimple’s Antibody Diluent 2. The secondary antibody and chemiluminescent substrate used the Anti-Rabbit Detection module (#DM-001; ProteinSimple, San Jose, CA, USA). Proteins were quantified and visualized by the “Compass for SW 5.01” software (ProteinSimple, San Jose, CA, USA).

### 2.10. Statistical Analysis

The results of the luciferase assay, ddPCR and Western blotting were generated by three biological replicates, which means iVLPs are produced by transfected cells from different wells or dishes.

Statistical analysis was conducted using GraphPad Prism 9.5 software, employing an unpaired *t*-test (assume Gaussian distribution) to compare between two groups. A significance level of *p* < 0.05 was considered indicative of a statistically significant difference and was represents as “*”, *p* < 0.01 was represents as “**”, and *p* < 0.001 was represents as “***”.

## 3. Results

### 3.1. Spatial Co-Localization of SARS-CoV-2 Packaging Signal with 5′/3′ UTRs

Building on the high-resolution RNA interactomes of SARS-CoV-2 generated by SPLASH and COMRADES [14,19], we performed a targeted reanalysis to decode the spatial interactions between defined bait regions and distal genomic elements.

The fragment PS9, located in nsp15 and nsp16 in the SARS-CoV-2 genome, was reported as the viral packaging signal [5].

**Figure 1:**
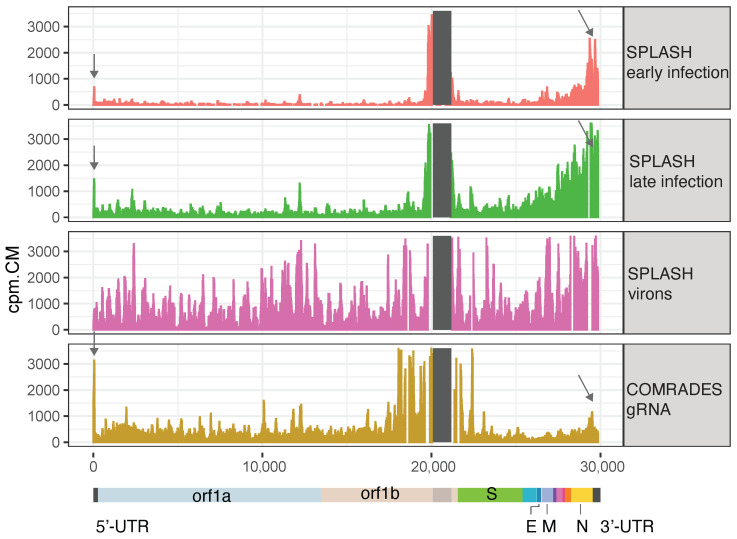
Proximity analysis of published SPLASH and COMRADES data. SPLASH data were captured in the early infected stage (red histogram), late stage (green histogram), and virions (magenta histogram). COMRADES data were captured in gRNA (ochre yellow). The dark gray rectangles indicate the location of PS9 and the arrows indicate the interactive sequences. The bottom panel shows a diagram of the SARS-CoV-2 genome to map the relative position of the locations.

Using PS9 as a sequence-specific bait, we systematically quantified its ligation frequency across the SARS-CoV-2 genome at three distinct stages of viral infection: (i) early infection (pre-cytopathic effect [CPE]), (ii) late infection (active CPE with 70% cellular involvement), and (iii) mature virions. Our SPLASH analysis revealed statistically significant UTR-PS9 interaction frequencies during both the pre-CPE and CPE stages, which were abolished in mature virions, corresponding to periods when viral genomes maintain relaxed conformations to facilitate replication and transcription processes. These interaction patterns were independently validated through COMRADES datasets, confirming the spatial proximity between UTRs and PS9 during intracellular replication phases (Figure 1).

### 3.2. 5′ UTR and 3′ UTR Enhance the iVLPs Genome Packaging

**Figure 2:**
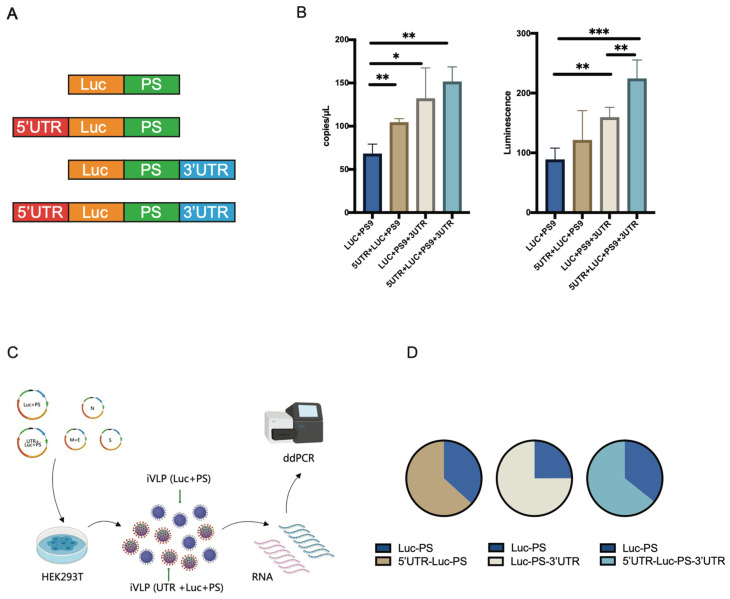
5′ UTR and 3′ UTR effect on SARS-CoV-2 packaging initiation. (**A**) Design of iVLP genomes. Luc, luciferase coding gene. PS, validated packaging signal PS9. 5′ UTR and 3′ UTR, 5′ UTR and 3′ UTR of SARS-CoV-2 genome. (**B**) Luciferase readout of different iVLP genomes’ packaged iVLPs. (**C**) Overview of the competition test of iVLP genomes with or without UTRs. (**D**) Proportions of different genome copies in iVLPs. Results of luciferase assay and ddPCR were generated by at least three biological replicates. Statistical analysis was conducted using an unpaired *t*-test to compare between two groups. A significance level of *p* < 0.05 was considered indicative of a statistically significant difference and was represents as “*”, *p* < 0.01 was represents as “**”, and *p* < 0.001 was represents as “***”.

Drawing inspiration from the work of Syed et al. [3], a virus-like particle genome was cloned to validate its function. It is shown that the iVLPs genome containing PS9, although much shorter than SARS-CoV-2, could be packaged into assembled viral structure proteins efficiently to form spherical particles of about 100 nm diameter, which is similar to the authentic viruses in terms of morphology and immunogenicity (Supplementary Figure S1). This iVLPs platform could serve as a simple model for dissecting coronavirus genome-packaging mechanisms. To precisely define the UTR functions in PS9-mediated packaging, we engineered a panel of modular iVLPs genomes with defined configurations: (i) PS9 core (baseline control, designated as “Luc-PS9”), (ii) PS9 + 5′ UTR (5UTR-Luc-PS9), (iii) PS9 + 3′ UTR (Luc-PS9-3UTR) and (iv) PS9 + 5′/3′ UTR dual configuration (5UTR-Luc-PS9-3UTR). All constructs were replication-incompetent (Figure 2A). These constructs were transfected alongside structural protein-coding genes, and the RNA from the supernatant of the resulting iVLPs was subjected to reverse transcription and droplet digital PCR (ddPCR) analysis. The iVLPs containing SARS-CoV-2 5′ UTR and/or 3′ UTR exhibited higher copy numbers than iVLPs lacking UTRs, indicating there were more viral particles in these samples (Figure 2B). Additionally, luciferase assays using ACE2-expressing HEK293 cells infected with RNaseA-treated iVLPs showed that the presence of UTRs significantly enhanced reporter gene expression, serving as an indicator of iVLPs’ infectivity, and aligning with the ddPCR findings (Figure 2B).

To quantitatively resolve the competitive advantage conferred by UTR elements in SARS-CoV-2 genome packaging, we implemented a pairwise competition assay wherein structural protein genes (N/S/M-E) were co-transfected with two distinct iVLPs genomes—one serving as a UTR-deficient reference (Luc-PS9) and the other as a UTR-containing test variant—under identical cellular conditions (Figure 2C). This experimental design enabled the direct comparison of packaging efficiency within a shared structural protein pool, eliminating expression-level variability. The genome copies in iVLPs or transfected cells were extracted and quantified by ddPCR analysis; then, the relative packaging efficiency could be calculated. Crucially, the primer specificity and amplification efficiency for each competitor genome were rigorously validated (Supplementary Figure S2 and Tables S1 and S2). We found that 5′ UTR and/or 3′ UTR could enhance packaging efficiency (Figure 2D and Figure S3). Collectively, these data establish UTRs as cis-regulatory elements that optimize packaging efficiency through competitive fitness enhancement, with the 3′ UTR exerting maximal functional potency.

### 3.3. 5′ UTR and 3′ UTR Recruit More Nucleocapsid Proteins in iVLPs

**Figure 3:**
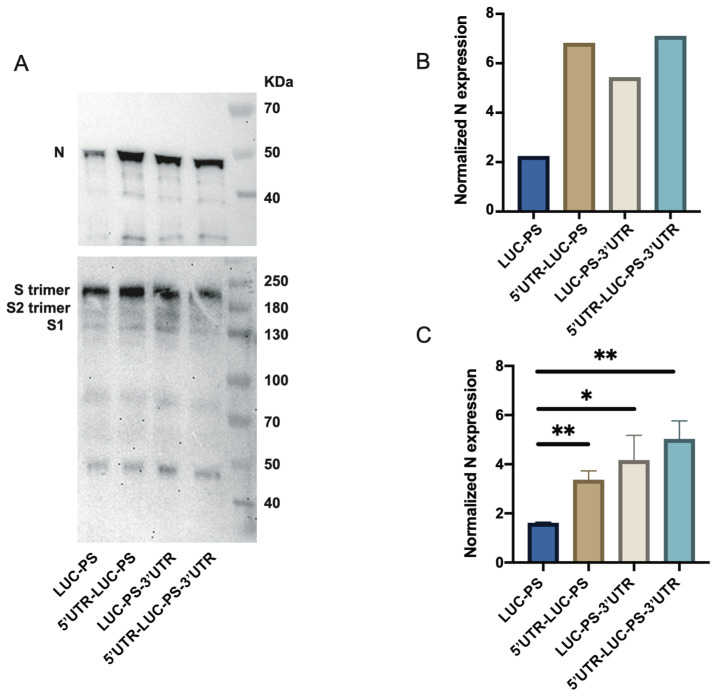
5′ UTR and 3′ UTR’s influence on relative N expression in iVLPs. (**A**) N and S protein expression in iVLPs, detected by Western blotting. (**B**) Relative N expression in VLPs, normalized by S expression. (**C**) Relative N expression in iVLPs, detected by digital Western blotting and normalized by S expression. Results of Western blotting were generated by three biological replicates. Statistical analysis was conducted using unpaired *t*-test to compare between two groups. A significance level of *p* < 0.05 was considered indicative of a statistically significant difference and was represents as “*” and *p* < 0.01 was represents as “**”.

It is known that the role of N proteins is specifically very important in the condensing and packaging of viral RNA [20]. We therefore hypothesized that incorporating UTRs into iVLPs genomes affects N protein encapsulation. To test this mechanistically, we quantified N protein incorporation in purified iVLPs via Western blotting, using the spike (S) protein as an internal reference due to its non-involvement in packaging signal interactions. Intriguingly, iVLPs carrying UTR elements demonstrated > 2-fold higher N protein incorporation compared to the Luc-PS9 control (Figure 3A,B). This observation was independently validated by digital Western analysis with triplicate biological replicates, which confirmed consistent > 2-fold N protein enrichment in UTR-containing iVLPs (*p* < 0.01 vs. Luc-PS9) (Figure 3C). Collectively, these data demonstrate that UTR elements augment nucleocapsid recruitment during iVLPs assembly, potentially through enhanced N protein oligomerization or LLPS-facilitated genomic condensation. This finding reinforces the critical role of N proteins as a trans-acting regulator in SARS-CoV-2 packaging initiation.

### 3.4. Identification of Core Sequence at 5′ UTR and 3′ UTR in Virus Packaging

**Figure 4:**
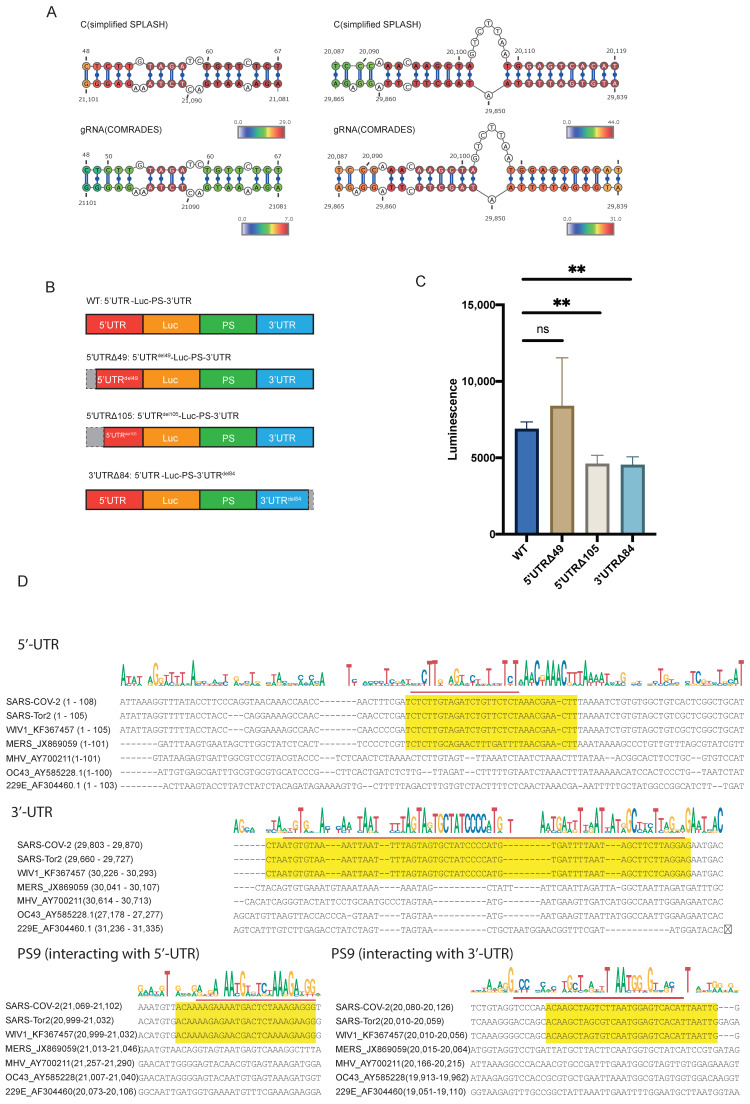
5′ UTR and 3′ UTR core sequence identification. (**A**) Base-paring analysis of UTRs and packaging signal in SPLASH and COMRADES data using Mfold. Heatmap indicates interaction frequency in the dataset of ligation proximity. (**B**) Design of iVLPs genomes containing full-length UTRs or truncated UTRs. (**C**) Luciferase readout of different genomes’ packaged iVLPs. (**D**) Multiple sequence alignment of the core regions within the packaging signal (PS9), 5′ and 3′ untranslated regions (UTRs) of SARS-CoV-2, and several other coronaviruses. The results of the luciferase assay were generated by three biological replicates. Statistical analysis was conducted using unpaired *t*-test to compare between the two groups. A significance level of *p* < 0.05 was considered indicative of a statistically significant difference and *p* < 0.01 was represents as “**”.

To elucidate the molecular mechanism underlying UTR-mediated packaging enhancement, we performed an integrated analysis combining computational predictions with experimental validation. Secondary structure predictions by Mfold, corroborated by SPLASH-derived interaction frequencies, identified two highly conserved RNA motifs critical for UTR-PS9 interactions: a ~20-nucleotide segment (nt 48–67) within the 5′ UTR SL2/3 and a ~30-nucleotide region (nt 29,839–29,865) in the 3′ UTR hypervariable domain. These elements exhibited Watson–Crick complementarity to PS9 segments 21,081–21,101 nt and 20,087–20,119 nt, respectively, possibly forming the stable base-pairing interactions essential for maintaining spatial proximity between UTRs and PS9 (Figure 4A).

To functionally validate these predictions, we engineered a series of truncated iVLPs genomes, including 5′ UTRΔ49 (retaining core interaction sequences), 5′ UTRΔ105 (deleting first 105 nt including the core motif), and 3′ UTRΔ84 (removing 84 nt containing the interaction sequences) (Figure 4B).

Quantitative analysis demonstrated that deletion of core interaction motifs (5′ UTRΔ105 and 3′ UTRΔ84) resulted in a significant reduction in packaging efficiency (*p* < 0.01), while the 5′ UTRΔ49 mutant maintained an activity comparable to the full-length control (5UTR-LUC-PS-3UTR) (Figure 4C). Pairwise competitive packaging assays further confirmed complete loss of selective advantage in 5′ UTRΔ105 mutants (Supplementary Figure S4). These findings underscore the importance of predicted Watson–Crick base pairing sequences located in the UTRs for the initiation of iVLPs genome packaging.

Strikingly, phylogenetic analysis revealed these interaction motifs are conserved exclusively among SARS-CoV-2, SARS-CoV, and WIV1 [21], while showing reduced sequence identity with MERS, MHV, HCoV-OC43 and HCoV-229E (Figure 4D). These findings establish that sequence-specific RNA–RNA interactions between UTR core elements and PS9 represent a conserved structural determinant of efficient genome packaging in coronaviruses.

## 4. Discussion

### 4.1. iVLPs System Is a Simplified Model for Authentic Viruses

Although the specific roles of the 5′ and 3′ UTRs in SARS-CoV-2, particularly in terms of gene translation and host–pathogen interactions, have been diversely interpreted [22,23,24,25], it has been suggested that UTRs lack the capacity to autonomously initiate viral particle packaging. The present study advances the understanding of the contribution of UTRs to SARS-CoV-2 iVLPs genome packaging. Initial analysis of SPLASH and COMRADES data derived from authentic SARS-CoV-2 revealed direct interactions between the 5′/3′ UTRs and the packaging signal PS9, prompting us to investigate the functional relevance of these contacts. To this end, we employed the iVLPs system as a simplified model to dissect the specific contributions of UTRs to genome packaging. It should be noted that iVLPs system is a simplified model that mimics certain aspects of coronavirus assembly. Although iVLPs display a similar morphology and diameter to authentic virions, the relatively short genome is obviously different. The iVLPs genome contains only a reporter gene and packaging signal, with or without UTRs, which is convenient for dissecting the function and interaction of these elements. However, this reduced model does not fully recapitulate the context of a complete viral genome, which possesses global genome organization, and several genome fragments have also been reported to exhibit packaging activity [5,6]. Therefore, the conclusions of our research are limited to the iVLPs system, but might provide useful insights into the packaging mechanism of authentic coronaviruses.

### 4.2. Temporal of UTR-PS9 Interactions Defines Packaging Initiation

Our study, using a simplified iVLPs model, unveils the intricate interaction between the SARS-CoV-2 packaging signal PS9 and 5′ and 3′ UTRs, adding a sophisticated layer to our understanding of the viral genome packaging. Reanalysis of proximity ligation data from the simplified SPLASH and COMRADES datasets suggests the active involvement of UTRs in the packaging mechanism, indicating that UTRs directly engage with the PS9 element during this process.

The proximity ligation data were captured from three stages in the virus life cycle, before CPE, after CPE, and viral particles, representing sequential phases after infection. Notably, long-distance interactions between PS9 and UTRs were significant in the first two stages, but were undetectable in mature virions. This dynamic suggests that UTR-PS9 contacts function as transient scaffolds facilitating early packaging events, such as genome folding, rather than maintaining the final condensed nucleocapsid structure. We propose this loss might stem from several non-mutually exclusive reasons: (i) extreme RNA compaction during maturation physically obscures specific RNA–RNA interfaces, rendering them inaccessible to crosslinking or ligation; (ii) these contacts are no longer required after packaging is completed; (iii) technical biases, including differences in crosslinking accessibility or ligation efficiency between free and packaged RNA, may contribute. This temporal pattern mirrors observations in HIV-1, where Gag specifically binds viral RNA packaging signals in infected cells, while mature virions exhibit nonspecific RNA binding due to genome compaction [1,25].

### 4.3. Mechanism of N Protein Enrichment to Packaging Efficiency

Our data demonstrate that the incorporation of N proteins is increased approximately 2-fold in SARS-CoV-2 iVLPs, whose genomes contain 5′ and/or 3′ UTRs (Figure 3A–C). This increase correlates with enhanced packaging efficiency (Figure 2B left and D) and infectivity (Figure 2B, right), consistent with the earlier study performed by Syed et al., which displayed a correlation between reporter gene expression and relative N protein levels in packaging cells [5].

It is widely accepted that the electrostatic interactions between positively charged N proteins and negatively charged RNA drive genome encapsulation [20]. Our data show that the inclusion of UTRs correlates to increased N protein incorporation and enhanced packaging efficiency. Two non-exclusive mechanistic possibilities may account for this observation. One possibility is that UTR–PS9 interactions remodel the genome into a conformation that is more efficiently condensed by N proteins, either by creating a more favorable substrate for N binding or by improving the overall packaging competence of the RNA. In this scenario, the increased N protein incorporation could be either a driver or a consequence of enhanced packaging efficiency. Another possibility, supported by previous studies demonstrating that the SARS-CoV-2 N protein undergoes liquid–liquid phase separation (LLPS) when exposed to viral RNA elements, including PS9, 5′ UTR, and 3′ UTR [9,11,12], is that UTRs facilitate packaging by promoting N protein recruitment through phase-separation-mediated condensation. Our current experimental data do not allow us to distinguish among these mechanistic scenarios, and further studies—such as real-time assembly kinetics or direct measurement of N–RNA binding affinity—are warranted to resolve the causal relationships.

### 4.4. The Interpretion of Infectivity Reduction in Truncates iVLPs Genomes

Through truncation assay, we closely mapped essential regions in UTRs that contribute to iVLPs reporter gene expression, which is consistent with the prediction of base pairs with PS9. This suggests that UTRs might contribute to coronavirus packaging by interacting with PS9. Since spatial proximity between RNA regions did not directly validate the base pairings predicted by Mfold, the reduction in the infectivity of truncated iVLPs genomes could reflect the disruption of Watson–Crick interactions or other local/global RNA secondary structures.

It is important to note that a previous study reported that the mere act of truncation does not account for the observed decrease in reporter gene transduction and expression [5,26]. The differential capacity of RNAs, despite similar lengths, to induce N protein condensation suggests that sequence and structure are pivotal in driving the assembly process. Our study, therefore, provides a nuanced understanding of the multifaceted roles of UTRs in the packaging of SARS-CoV-2 iVLPs, offering insights that could inform the development of targeted antiviral strategies.

## Data Availability

The data presented in this study are included in the article and Supplementary Material. Further inquiries can be directed to the corresponding authors.

## Supplementary Materials

The following supporting information can be downloaded at: https://www.mdpi.com/article/10.3390/v18070700/s1, Figure S1: Morphology and immunogenicity of iVLPs; Figure S2: Validation of primers specificity and amplification efficiency; Figure S3: Proportions of different genome copies in iVLPs with 3 biological replicates; Figure S4: Pairwise competitive packaging assays of truncated mutants; Table S1: the primer sequences for each competitor genome; Table S2: the primer amplification efficiency for each competitor genome.

## Abbreviations

The following abbreviations are used in this manuscript:

UTRs	untranslated regions
iVLP	infectious virus-like particle
gRNA	genomic RNA
sgRNA	subgenomic viral RNA
ssRNA	single-stranded RNA
RNP	beads-on-string ribonucleoprotein
PSs	packaging signals
SPLASH	sequencing of psoralen crosslinked, ligated, and selected hybrids
COMRADES	crosslinking of matched RNAs and deep sequencing
ddPCR	droplet digital PCR
CPE	cytopathic effect
