# Supplementary material for: Involvement of 5′ and 3′ UTRs in SARS-CoV-2 Virus-like Particle Genome Packaging
Source: Viruses. 2026 Jun 25;18(7):700. doi: 10.3390/v18070700 (PMC13431415; doi:10.3390/v18070700)
Supplement: Supplementary file 1 [file viruses-18-00700-s001.zip › Supplementary table-S1 S2.pdf]

Supplementary table S1

Primers	Sequence
Luc-PS-3UTR fwd	GCTGGCTAGCTTGTTAGACGAAGCTTGCCGCCATGGAAGATGCC
Luc-PS-3UTR rev	CTCCTTCTTAAAGGAGGCGGCC
tru-5UTR-1 fwd	CTGGCTAGCTTGTTAGACGAAGCTTCTTGTAGATCTGTTCTCTA
tru-5UTR-2 fwd	CTGGCTAGCTTGTTAGACGAAGCTTCATGCTTAGTGCACTCAC
tru-5UTR rev	CTGGCTAGCTTGTTAGACGAAGCTTCATGCTTAGTGCACTCAC
5UTR fwd	CGGGTGTGACCGAAAGGTAA
5UTR rev	GGGCCCTTCTTAATGTTTTTGG
5UTR prb	ACCGGTCGCCGCCATGGA
3UTR fwd	GGACTTGAAAGAGCCACCACAT
3UTR rev	CATTGTTCACTGTACACTCGATCGT
3UTR prb	TTCACCGAGGCCACGCGGA
Luc fwd	CTGGCTAGCGCCGCC
Luc rev	CCGTCTTCGAGTGGGTAGAATG
Luc prb	ATGCCAAAAACATTAAGAAGGGCCCAGC

Supplementary table S2

	Luc-PS	5'UTR-Luc-PS	Luc-PS-3'UTR	5"UTR-Luc-PS-3'UTR
Equation	Y = -3.030*X + 6.926	Y = -2.800*X + 17.36	Y = -2.221*X + 24.85	Y = -3.685*X + 16.39
