# Supplementary figures and images for: Involvement of 5′ and 3′ UTRs in SARS-CoV-2 Virus-like Particle Genome Packaging
Source: Viruses. 2026 Jun 25;18(7):700. doi: 10.3390/v18070700 (PMC13431415; doi:10.3390/v18070700)

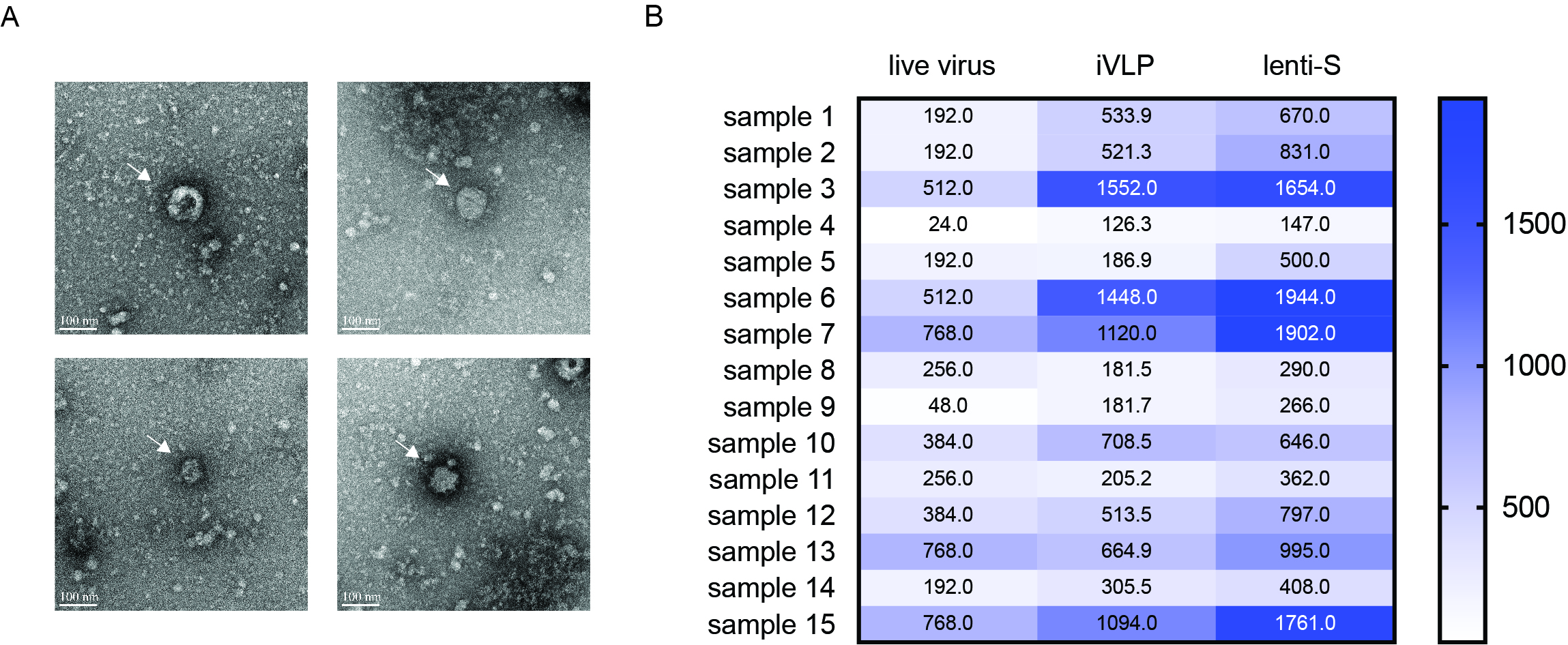

Supplement: Supplementary file 1 [file viruses-18-00700-s001.zip › Supplementary Fig-S1.jpg]

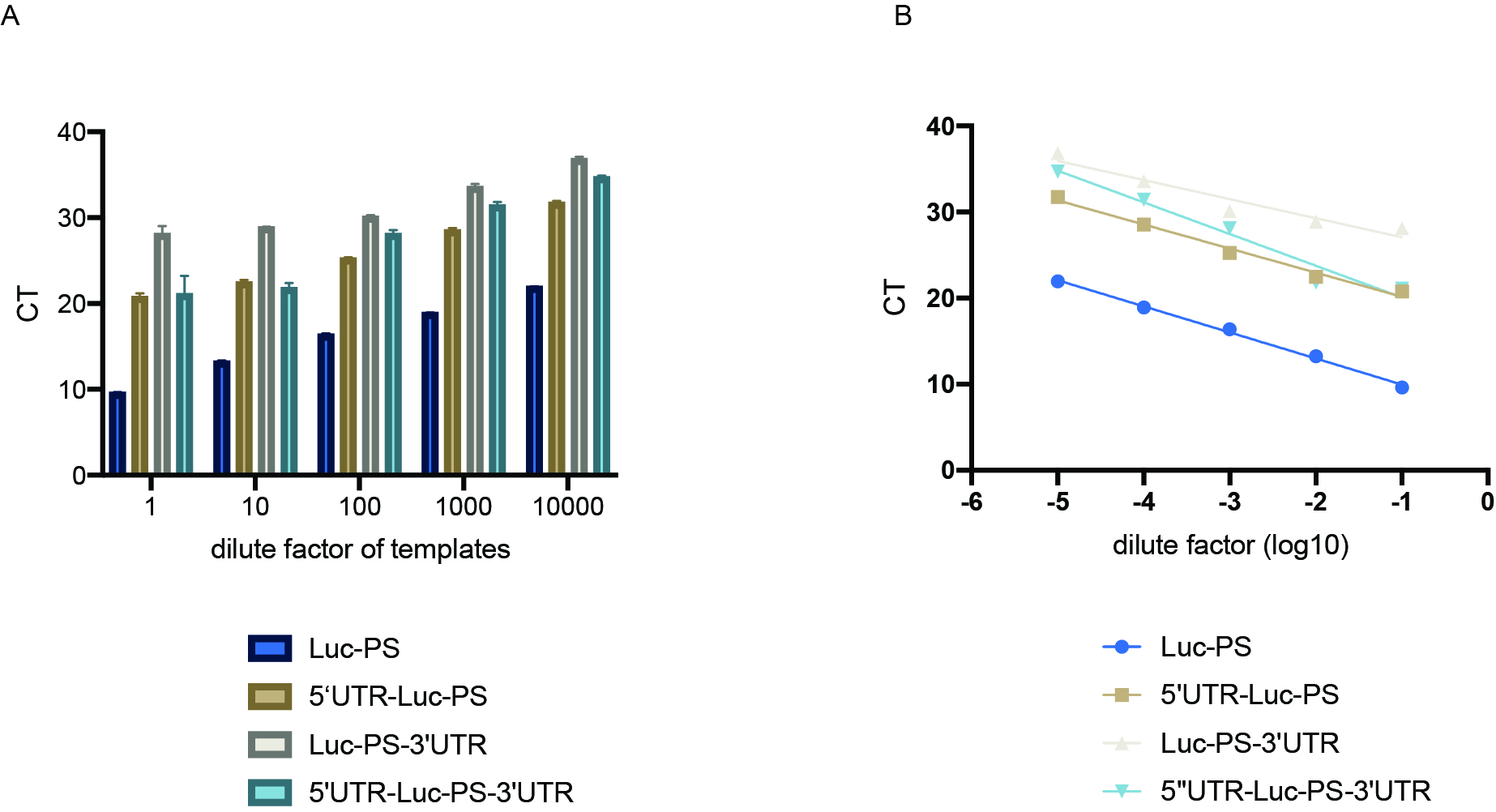

Supplement: Supplementary file 1 [file viruses-18-00700-s001.zip › Supplementary Fig-S2.jpg]

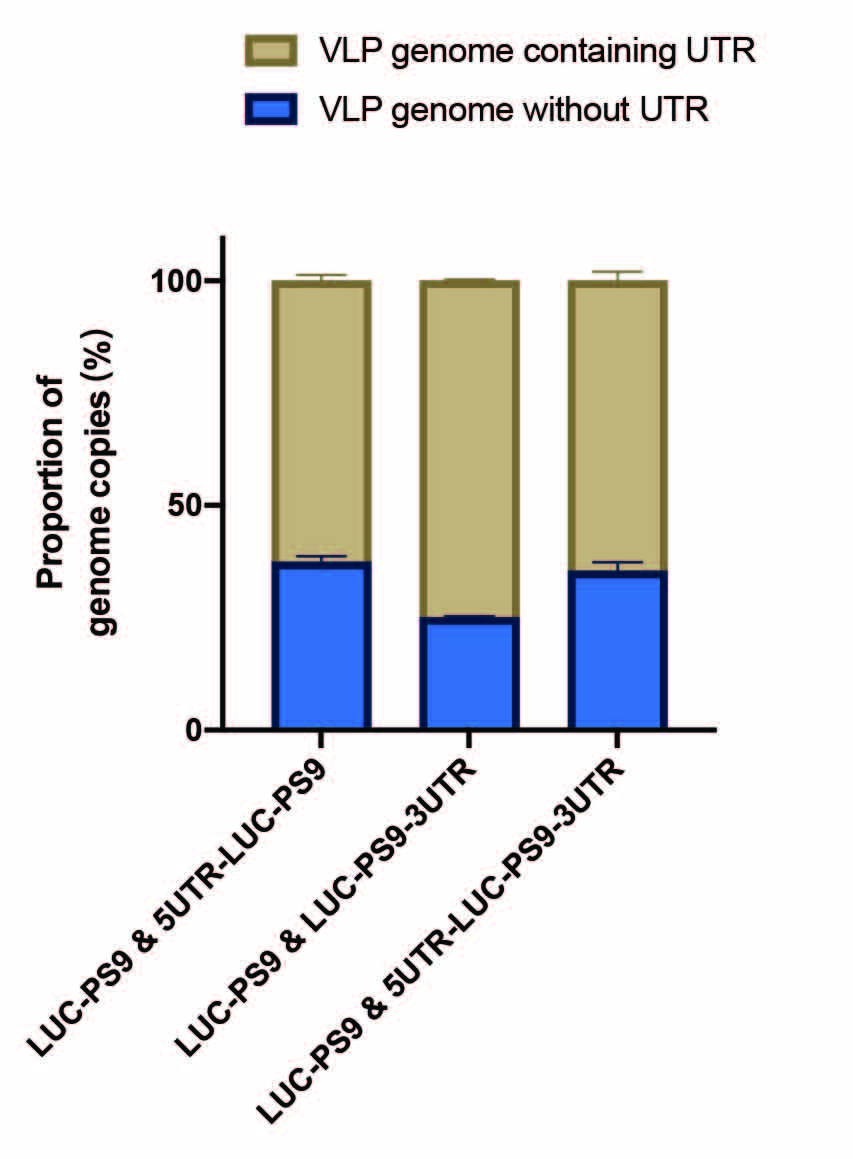

Supplement: Supplementary file 1 [file viruses-18-00700-s001.zip › Supplementary Figure-S3 20260625.jpg]

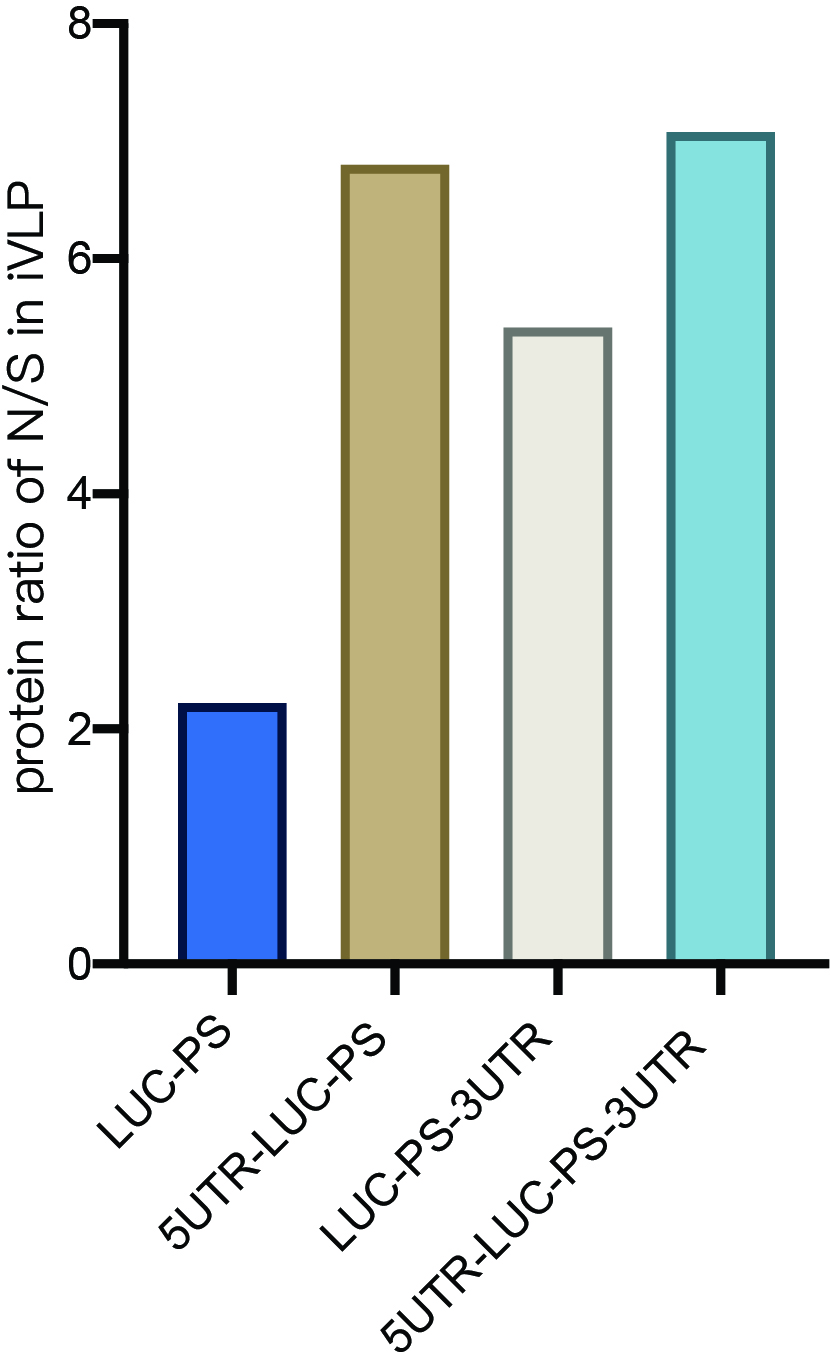

Supplement: Supplementary file 1 [file viruses-18-00700-s001.zip › Supplementary Figure-S4.jpg]
